# Differences in clinical presentation and management between pre- and postsurgical diagnoses of urinary bladder paraganglioma: is there clinical relevance? A systematic review

**DOI:** 10.1007/s00345-021-03851-x

**Published:** 2021-10-16

**Authors:** Minghao Li, Xiaowen Xu, Nicole Bechmann, Christina Pamporaki, Jingjing Jiang, Stefan Propping, Longfei Liu, Johan F. Langenhuijsen, Karel Pacak, Graeme Eisenhofer, Jacques W. M. Lenders

**Affiliations:** 1 Department of Medicine III, Technische Universität Dresden, Dresden, Germany; 2 Department of Urology, Xiangya Hospital, Central South University, Changsha, China; 3 Institute of Clinical Chemistry and Laboratory Medicine, Technische Universität Dresden, Dresden, Germany; 4 Department of Endocrinology and Metabolism, Zhongshan Hospital Fudan University, Shanghai, China; 5 Department of Urology, Technische Universität Dresden, Dresden, Germany; 6 Department Urology, Radboud University Medical Center, Nijmegen, The Netherlands; 7 Section on Medical Neuroendocrinology, Eunice Kennedy Shriver National Institute of Child Health and Human Development, National Institutes of Health, Bethesda, MD USA; 8 Department of Internal Medicine, Radboud University Medical Center, Geert Grooteplein Zuid 8, PO Box 6500 HB, 6525 GA Nijmegen, The Netherlands

**Keywords:** Diagnosis, Paraganglioma, Treatment, Urinary bladder

## Abstract

**Purpose:**

Paraganglioma of the urinary bladder (UBPGL) is a rare neuroendocrine tumor diagnosed in many patients only after surgery. We, therefore, assessed clinical clues relevant to presurgical diagnosis and clinical consequences in patients with a missed presurgical diagnosis of UBPGL.

**Materials and methods:**

Case reports describing a UBPGL (published from 1–1–2001 and 31–12–2020) were identified in Pubmed. Two authors independently performed data extraction and assessed data quality according to the PRISMA guideline. Patients were divided into two groups: UBPGL diagnosis before and after surgery.

**Results:**

We included 177 articles reporting 194 cases. In 90 (46.4%) patients, the UBPGL was diagnosed before and in 104 (53.6%) after surgery. In presurgically diagnosed UBPGL, hypertension and catecholamine-associated symptoms were 2- to 3-fold (*p* < 0.001) more frequent than in postsurgically diagnosed patients whereas hematuria was twofold (*p* = 0.003) more prevalent in those with postsurgical diagnosis. Hypertension was an independent factor for presurgical biochemical testing (OR 4.45, 95% CI 1.66–11.94) while hematuria (OR 0.23, 95% CI 0.09–0.60) was an independent factor for not performing presurgical biochemical testing. Most patients diagnosed after surgery were not pretreated with alpha-adrenoceptor blockade (95.2%), underwent more frequently transurethral resection instead of cystectomy (70.2% vs. 23.1%) and had more frequent peroperative complications and residual tumor mass.

**Conclusions:**

In nearly half of all patients with a UBPGL, the diagnosis was not established before surgery. Hypertension and hematuria contributed independently to a presurgical diagnosis. Postsurgical diagnosis, which was associated with suboptimal presurgical and surgical management, resulted in more peroperative complications and incomplete tumor resections.

**Supplementary Information:**

The online version contains supplementary material available at 10.1007/s00345-021-03851-x.

## Introduction

Paragangliomas are neuroendocrine tumors that originate in extra-adrenal paraganglia [1]. A paraganglioma of the urinary bladder (UBPGL) is particularly rare, accounting for only 0.05% of all bladder tumors [2]. Its origin likely reflects development from paraganglia embedded in the muscular layer of the bladder wall [3]. Patients with UBPGL may show signs and symptoms caused by episodic secretion of catecholamines triggered by micturition. These include paroxysms of high blood pressure (BP), headache, sweating, palpitations, and nausea.

There is no specific guideline for surgical treatment of UBPGL. In general, standard treatment for muscle-invasive tumors is partial or radical cystectomy while bladder tumors that are not muscle-invasive can be treated with transurethral resection (TUR) [4–6]. Since UBPGLs seem to originate from within the muscle layer of the urinary bladder, the preferred treatment of UBPGLs is either partial or radical cystectomy.

Irrespective of the mode of surgery, the presence of a catecholamine-producing UBPGL requires preoperative alpha-adrenoceptor blockade to mitigate the risk of a catecholaminergic crisis that may follow tumor manipulation [7]. Such crisis usually include severe derailment of BP and heart rhythm, potentially culminating in life-threatening cardiovascular complications during surgery. Therefore, a correct presurgical diagnosis is pivotal to decisions concerning initiation of presurgical blockade of alpha-adrenoceptors and for prevention of serious peroperative cardiovascular complications [8].

A presurgical diagnosis of UBPGLs is challenging due to the rare nature of the tumor and its associated high risk that clinical clues may be overlooked [9]. The clinical clues for diagnosis of the tumor include paroxysmal hypertension and catecholamine-associated symptoms (CAS) related to micturition. Outside micturition, BP may be normal and patients may be asymptomatic [10, 11]. Once a UBPGL is considered, the biochemical diagnosis is now straightforward and includes measurements of metanephrines (metabolites of catecholamines) in plasma or urine [8, 12].

Since the report by Zimmerman et al. on UBPGL in 1953 [13], about 250–300 case reports and case series have been published; in many patients, the diagnosis was not made before initial surgery [9]. As the underlying reasons for not making the diagnosis before surgery are not entirely clear, we systemically reviewed all cases and case series on UBPGL that were reported over the past 20 years. The main purpose of this study was to evaluate the differences in clinical presentation, presurgical diagnostic workup and the peroperative management between patients diagnosed before versus after surgery and to assess the clinical consequences of an overlooked diagnosis before surgery.

## Materials and methods

### Data sources and search strategy

Two researchers (ML and XX) independently searched PubMed for articles published in English or Chinese language from 1–1–2000 to 31–12–2020. The following search terms were used: ((pheochromocytoma [MeSH Terms]) OR (pheochromocytoma [Title/Abstract]) OR (paraganglioma [Title/Abstract])) AND ((bladder [MeSH Terms]) OR (bladder [Title/Abstract])).

### Study selection and data extraction

Based on title and abstract, ML and XX independently selected the papers that reported on patients with UBPGLs. Subsequently, full text papers were downloaded if available. Articles without extractable data of individual cases were excluded as were cases without an explicit clear description about whether the diagnosis of UBPGL was made before or after surgery (Figure S1). ML and XX independently accessed all cases and extracted items according to the definitions (Table S1). Items not explicitly reported were noted as ‘not done’ or ‘not available’. This study was performed according the PRISMA guideline [14].

### Data analysis

The diagnosis of UBPGL was defined by positive results of biochemical testing or by pathological evidence in tissue specimens obtained by biopsy or surgery. Patients were categorized into two different groups according to the time of diagnosis: (1) presurgical diagnosis: in patients with confirmed diagnosis before first surgery; (2) postsurgical diagnosis: in patients with a confirmed diagnosis of UBPGL established after first surgery, usually by histopathological examination of removed tissue.

We analyzed differences between the two groups in clinical characteristics, presenting signs and symptoms and biochemical testing. We also evaluated whether a diagnosis before or after first surgery would have led to differences in treatment (installment of presurgical alpha-adrenoceptor blockade, type of surgical approach), in surgical course (intraoperative complications such as derailment of BP, cardiovascular incidents, discontinued surgery) or postoperative residual tumor.

### Statistical analysis

IBM SPSS statistics 25 (Systat Software GMbH, Erkrath, Germany) was used for data analysis. Categorical variables are presented as numbers and percentages. Continuous data that were normally distributed are presented as mean ± SDs and non-normally distributed data are expressed as medians and interquartile ranges (IQR). The Student *t* test was used for normally distributed continuous data whereas Mann–Whitney test was used for non-normally distributed continuous data. Chi-square or the Fisher’s exact tests were used to compare categorical data. Binary logistic regression was used for identification of variables related to use of biochemical tests before surgery. For those variables that were significantly different in the univariable analysis, we performed multivariable logistic regression analysis to identify independent factors that affected performing of biochemical testing. Odds ratios (ORs) and 95% confidence intervals (CI) were reported. *p* values less than 0.05 were considered statistically significant.

## Results

### Search results and study selection

Three hundred and forty articles were identified in Pubmed (Figure S1). We excluded 113 articles after review of titles and abstracts. Articles of which full text papers were not available or without extractable detailed data on individual cases were additionally excluded (*n* = 30). Among the 301 cases extracted from 197 articles, 107 cases were excluded because details on pre- or postsurgical diagnosis were not available. Finally, 194 cases from 177 articles were included for final analysis (Figure S1) (Table S2).

### Characteristics of all patients with UBPGL

Patients (98 female, 94 male and 2 with unknown gender) with UBPGL presented to hospitals at ages from 7 to 81 years. One hundred and sixteen patients presented with CAS and/or hypertension (Table S3, Figure S2), including 95 with hypertension and 87 patients who had 1 or more CAS. The latter were triggered by micturition in 59 patients (Table S3). Thirty-six patients presented with only hematuria before surgery (Figure S2), although several other patients with CAS and/or hypertension also had hematuria. Twenty-two patients presented with other signs and symptoms such as pelvic or lower abdominal pain, and 20 patients were reported to have no signs and symptoms.

Sixty-three of 78 patients who underwent biochemical testing had positive test results (Figure S3). In 23 of 26 patients who had both biochemical testing and a tumor biopsy, a positive result of biochemical testing and/or biopsy was reported. In 4 of 13 patients who underwent a tumor biopsy alone, the results indicated a UBPGL.

The nature of initial surgical procedures was reported in 173 patients, 95 of whom underwent partial or total cystectomy and 78 underwent TUR (Table S3, Figure S2). In 28 (14.4%) patients, peroperative systolic BP exceeded 180 mmHg and in 10 (5.2%), a cardiovascular incident was reported during surgery. This resulted in discontinuation of surgery in 14 (7.2%) patients. After initial surgery, tumor removal was incomplete in 33 (17%) patients (Table S3). Discontinued surgery or residual tumor mass occurred more frequently in patients having TUR (42, 53.8%) than in those having cystectomy (3, 3.2%) (Figure S2).

### Clinical and diagnostic features of patients with pre- versus postsurgically diagnosed UBPGL

In 90 (46.4%) patients, the tumor was diagnosed as UBPGL before first surgery while in 104 (53.6%) patients the tumor was diagnosed after surgery. Patients with presurgically diagnosed UBPGL were younger than those diagnosed after surgery (Table S3). Compared to those with a postsurgical diagnosis, patients with presurgically diagnosed UBPGL presented with higher systolic (184.7 ± 45.8 vs. 138 ± 31 mmHg, *p* < 0.001) and diastolic BP (108.9 ± 23.5 vs. 85.8 ± 18.9 mmHg, *p* < 0.001). Accordingly, there was a nearly threefold higher frequency of hypertension (75.6% vs. 26%, *p* < 0.001) in patients with a pre- than postsurgical diagnosis. Similarly, the proportion of patients with CAS who had a presurgical diagnosis of UBPGL was higher (67.8%) than those who had a postsurgical diagnosis (25%) (*p* < 0.001). This applied also to the most relevant symptoms such as sweating, headache and palpitations (Table S3). In addition, patients who presented with CAS triggered by micturition had more frequently a presurgical than postsurgical diagnosis of UBPGL (51.1% vs. 12.5%, *p* < 0.001). In contrast, patients with hematuria had more often a postsurgical than a presurgical diagnosis of UBPGL (44.2% vs 23.3%, *p* = 0.003), (Table S3).

### Treatment of patients with pre- versus postsurgical diagnosed UBPGL

Presurgical preparation using alpha-adrenoceptor blockade was initiated in 53.3% of the patients diagnosed before surgery compared to only 4.8% of those with postsurgically diagnosed UBPGL (Table S3).

The initial surgical approach was significantly different between patients with a presurgical versus those with a postsurgical diagnosis (Table S3). In patients with a UBPGL diagnosed before surgery, 71 (78.9%) were treated by partial or total cystectomy and 5 (5.6%) had a TUR. In contrast, if the diagnosis was not established before surgery, most patients (73, 70.2%) underwent TUR and only 24 (23.1%) had cystectomy.

During surgery, systolic BP exceeded 180 mmHg in only three patients (3.3%) after a diagnosis was made before surgery (Table S3). This contrasted to 25 (24%) patients who were not pretreated as the diagnosis was made after surgery (*p* < 0.001) (Table S3). Remarkably, 19 of these 25 patients were not known to have hypertension before surgery. No single patient experienced a peroperative cardiovascular incident when diagnosed before surgery, while this occurred in ten (9.6%) patients with postsurgically diagnosed UBPGL, all without appropriate presurgical preparation. These differences between the groups translated to numbers of operations that had to be discontinued; specifically, in only 1 (1.1%) patient the operation had to be discontinued when the tumor was diagnosed presurgically compared to 13 (12.5%) patients with a postsurgical diagnosis (*p* = 0.004) (Table S3).

Median tumor size in patients with presurgically diagnosed UBPGL was 3.7 (IQR 2.6–5.0) cm, which was significantly larger than 2.7 (IQR 2.0–3.7) cm in those with postsurgically diagnosed UBPGL (*p* < 0.001). The proportion of patients with residual tumors was 4.8-fold higher in patients with postsurgically than presurgically diagnosed UBPGL; this mainly reflected the choice of TUR in patients in whom the diagnosis was not known before surgery (Table S3).

We also analyzed differences in treatment results between patients who had positive biochemical tests compared to those who had no biochemical testing (Table S4). Patients with positive biochemical test results had more often presurgical alpha-adrenoceptor blockade and more often cystectomy instead of TUR than patients who had no biochemical testing. In contrast, they had less frequently derailed systolic BP, peroperative cardiovascular incidents, discontinued surgeries and residual tumor mass than patients who had no biochemical testing.

### Factors determining the biochemical diagnosis of UBPGL before surgery

Univariable logistic regression analysis showed that age, tumor size, signs and symptoms including hypertension, CAS, hematuria and CAS triggered by micturition were found to be significantly related to the probability of undergoing presurgical biochemical testing (Table S5). After multivariable analysis, only younger age (OR 0.97, 95% CI 0.94–0.99, *p* < 0.05) and presentation with hypertension (OR 4.45, 95% CI 1.66–11.94, *p* < 0.05) were found to be independently associated with presurgical biochemical testing. In contrast, any presentation with hematuria made presurgical biochemical testing less likely as the OR was 0.23 (95% CI 0.09–0.60, *p* < 0.05) (Table S5).

## Discussion

This large systematic review in patients with a UBPGL demonstrates that the diagnosis of this rare bladder tumor is often not made until after surgery. This delayed diagnosis has several relevant clinical consequences. Patients with a postsurgical diagnosis have more cardiovascular incidents and more derailed systolic BP during surgery and as expected, this results in more discontinued surgeries compared to patients with a presurgical diagnosis. The increased proportion of intraoperative complications in those with no presurgical diagnosis likely reflects increased tumoral secretion of catecholamines during surgical procedures performed without alpha-adrenoceptor blockade. Finally, because more patients underwent tumor resection by TUR instead of cystectomy, more patients had residual tumor after surgery. It can be inferred from these data that a timely presurgical diagnosis would have resulted in less potentially harmful consequences for these particular patients.

A timely diagnosis of a UBPGL before surgery is crucial as this has implications for proper preparation of patients by administration of alpha-adrenoceptor blockers and choice of the most appropriate surgical approach. However, for a presurgical diagnosis of a UBPGL, the first requirement is early consideration of this tumor in patients who present with a urinary bladder mass. As can be inferred from the large number of patients in whom the diagnosis was not made until after surgery, it seems that the possibility that the bladder mass was a UBPGL was not considered. Lack of suspicion that a bladder mass could be a UBPGL is partly due to the low prevalence of these tumors as they account for only 0.05% of all bladder tumors while at the other end of the spectrum urothelial cell carcinoma account for more than 90% of all bladder tumors [2, 15]. This explains partially why in many cases the diagnosis was not made before surgery.

Another clue for the presence of a UBPGL can be derived from signs and symptoms of catecholamine excess, but this requires a meticulously taken medical history and physical examination. A clear differentiating sign in our study was hypertension, which was nearly threefold more frequently reported in patients with a presurgical than a postsurgical diagnosis, and which appeared to be a strong and significant independent factor (OR 4.45) that apparently triggered urologists to perform presurgical biochemical testing in the former group. Hypertension is, however, not specific for a catecholamine-producing tumor such as a UBPGL, as it may be a feature in a variety of clinical conditions associated with elevated sympathetic activity. However, in patients who present with an adrenal or bladder tumor and who have hypertension, a catecholamine-producing tumor should be considered and appropriate biochemical for catecholamine excess should be ordered. Patients with a presurgical diagnosis were reported to have a nearly 2.5-fold higher frequency of CAS than those with a postsurgical diagnosis. However, it cannot be ascertained from these data whether all patients with a postsurgical diagnosis were subjected to detailed interrogation on CAS. This also applied to CAS triggered by micturition, which was nearly four times more prevalent in patients with a presurgical than postsurgical diagnosis. Therefore, awareness of hypertension and of specific CAS such as sweating, headache and palpitations, with or without relation to micturition, will help urologists to obtain a presurgical diagnosis more frequently in patients with a UBPGL.

One other specific relevant symptom appeared to be hematuria, the most common symptom in patients with a bladder tumor (85%) [16]. The nearly twofold higher frequency of hematuria in patients with a postsurgical than presurgical diagnosis and this was even more so in patients reported with only hematuria. This was corroborated by the finding that hematuria was a significant and strong independent predictor (OR 0.23) of not engaging in biochemical testing for a UBPGL. The finding that nearly one-third of all patients with a UBPGL had hematuria indicates that this symptom cannot assist in excluding a UBPGL. This conclusion is supported by several other studies [2, 9, 17].

One important implication of our study is that biochemical testing for a catecholamine-producing urinary bladder paraganglioma by measurements of plasma metanephrines should be carried out in all patients who present with a bladder tumor and who are known with hypertension.

In this study, the diagnostic yield of a positive biochemical test for a presurgical diagnosis was nearly 2.5-fold higher (63/78, 80.8%) than that of a tumor biopsy (4/13, 30.8%), which supports that measurements of catecholamine metabolites and not a biopsy as the first method of choice for diagnosis. An additional drawback of tumor biopsy in patients with an unrecognized UBPGL includes the risk of a catecholaminergic crisis [18]. In addition, as UBPGLs are derived from paraganglia in the muscular layer of the bladder wall, insufficient deep biopsies limited to the mucosa may, therefore, result in missing the UBPGL or in misdiagnosis of urothelial cell carcinoma [3, 9, 19].

A crucial question involves the potentially harmful impact of a missed diagnosis of UBPGL before surgery. The current study is the first that strongly suggests that this is indeed the case. By not establishing the diagnosis before surgery, the result is omission of presurgical alpha-adrenoceptor blockade, as was the case in more than 95% of the patients with a postsurgical diagnosis [8]. Therefore, as expected, peroperative cardiovascular incidents occurred only in those who were not pretreated as they were not diagnosed before surgery. Together with nearly eightfold more systolic BP derailments in those with a postsurgical than presurgical diagnosis, more surgeries had to be discontinued. It is likely that more presurgical diagnoses of this tumor with proper pretreatment would have limited most of these unwanted sequelae.

A correct presurgical diagnosis of UBPGL impacts also on the optimal surgical approach of this tumor and our data confirm that the choice of partial or total cystectomy as the preferred surgical approach depends on a correct presurgical diagnosis.

## Strengths and limitations of the study

Our review covers the largest cohort of patients with UBPGLs published to date. Another strength of this study is that we included not only patients in whom the diagnosis was made before surgery but also those in whom the diagnosis was not considered before surgery. This enabled a more realistic assessment of the potential negative consequences of not establishing the diagnosis before surgery.

One of the limitations of our study is that a review of case reports and very small case series cannot provide the highest level of clinical evidence. However, we included a large cohort of cases and evaluated these as rigorous as possible. Another limitation is the retrospective nature of the study. This makes it impossible to ascertain whether all patients were questioned systematically about signs and symptoms. Another limitation is that we are not able to specify the potential role of imaging, especially functional imaging modalities, for the presurgical diagnosis. Both CT and MRI have some specific imaging characteristics supporting the diagnosis of a paraganglioma [20]. Similarly, it was not feasible to specify the diagnostic role of macroscopic findings at cystoscopy as most case reports did not describe the specific cystoscopy findings [9, 21, 22].

## Conclusions

Our study shows that slightly more than half of patients with a UBPGL are not diagnosed before initial surgery. This can be explained by not considering the diagnosis upfront despite the presence of hypertension and CAS. Hematuria should not be used to discard the possibility of a UBPGL. The consequences of an overlooked diagnosis before surgery are clinically relevant in terms of more peroperative cardiovascular incidents, more discontinued surgeries and more incomplete tumor resections than in patients with a presurgical diagnosis. Careful initial consideration of a potential UBPGL may result in more patients with a presurgical diagnosis and appropriate presurgical treatment, thereby avoiding peroperative complications and unnecessary reoperations.

## Supplementary Information

Below is the link to the electronic supplementary material.

Supplementary file1 (DOCX 265 KB)
