# Correction to: Differences in clinical presentation and management between pre- and postsurgical diagnoses of urinary bladder paraganglioma: is there clinical relevance? A systematic review

**DOI:** 10.1007/s00345-021-03888-y

**Published:** 2021-11-18

**Authors:** Minghao Li, Xiaowen Xu, Nicole Bechmann, Christina Pamporaki, Jingjing Jiang, Stefan Propping, Longfei Liu, Johan F. Langenhuijsen, Karel Pacak, Graeme Eisenhofer, Jacques W. M. Lenders

**Affiliations:** 1 Department of Medicine III, Technische Universität Dresden, Dresden, Germany; 2 Department of Urology, Xiangya Hospital, Central South University, Changsha, China; 3 Institute of Clinical Chemistry and Laboratory Medicine, Technische Universität Dresden, Dresden, Germany; 4 Department of Endocrinology and Metabolism, Zhongshan Hospital Fudan University, Shanghai, China; 5 Department of Urology, Technische Universität Dresden, Dresden, Germany; 6 Department Urology, Radboud University Medical Center, Nijmegen, The Netherlands; 7 Section on Medical Neuroendocrinology, Eunice Kennedy Shriver National Institute of Child Health and Human Development, National Institutes of Health, Bethesda, MD USA; 8 Department of Internal Medicine, Radboud University Medical Center, Geert Grooteplein Zuid 8, PO Box 6500 HB, 6525 GA Nijmegen, The Netherlands

## Correction to: World Journal of Urology 10.1007/s00345-021-03851-x

In the original publication of the article, the first name of the eighth author was incorrect. The correct name of the author should be as given below:

First Name: Johan F.

Last Name: Langenhuijsen.

The original article has been updated accordingly.

