# Supplementary material for: Differences in clinical presentation and management between pre- and postsurgical diagnoses of urinary bladder paraganglioma: is there clinical relevance? A systematic review
Source: World J Urol. 2021 Oct 16;40(2):385–90. doi: 10.1007/s00345-021-03851-x (PMC8921018; doi:10.1007/s00345-021-03851-x)
Supplement: Supplementary file 1 — Supplementary file1 (DOCX 265 KB) [file 345_2021_3851_MOESM1_ESM.docx]

**Differences in clinical presentation and management between pre- and postsurgical diagnoses of urinary bladder paraganglioma: is there clinical relevance?**

**a systematic review**

Minghao Li ^a,b^, Xiaowen Xu ^b^, Nicole Bechmann ^a,c^, Christina Pamporaki ^a^, Jingjing Jiang ^d^, Stefan Propping ^e^, Longfei Liu ^b^, Hans Langenhuijsen ^f^, Karel Pacak ^g^, Graeme Eisenhofer ^a,c^, Jacques W. M. Lenders ^a,h*^

^a^ Department of Medicine III, Technische Universität Dresden, Dresden, Germany;

^b^ Department of Urology, Xiangya Hospital, Central South University, Changsha, China;

^c^ Institute of Clinical Chemistry and Laboratory Medicine, Technische Universität Dresden, Dresden, Germany;

^d^ Department of Endocrinology and Metabolism, Zhongshan Hospital Fudan University, Shanghai, China;

^e^ Department of Urology, Technische Universität Dresden, Dresden, Germany;

^f^ Department Urology, Radboud University Medical Center, Nijmegen, The Netherlands;

^g^ Section on Medical Neuroendocrinology, *Eunice Kennedy Shriver* National Institute of Child Health and Human Development, National Institutes of Health, Bethesda, Maryland, US;

^h^ Department of Internal Medicine, Radboud University Medical Center, Nijmegen, The Netherlands

^*^Correspondence to Jacques W.M. Lenders, MD, PhD, Radboud University Medical Center, Nijmegen, Department of Internal Medicine, P.O. Box 6500 HB, Geert Grooteplein Zuid 8, Nijmegen, 6525GA, The Netherlands.

Tel: +31243618819; fax: +31243541734; e-mail: [Jacques.lenders@radboudumc.nl](mailto:Jacques.lenders@radboudumc.nl)

**ORCID:** ML: 0000-0001-9267-0471; NB: 0000-0002-6932-333X

**Table S1.** Items for data extraction and definitions.

| Items | Definitions/Explanations | Outcome and measurement units |
| --- | --- | --- |
| Paper number | Number of paper | Number |
| Case number | Number of a case | Number |
| Author | First author | Name of first author |
| Journal | Name of journal | Abbreviated name of journal |
| Year | Year of publication | Year |
| Sex | Gender of patient | M/F/not reported |
| Age | Age at diagnosis | Number (years) |
| Primary UBPGL | Diagnosed as primary UBPGL. yes: if reported or if clear from description; no: if reported as absent | Yes/no/not reported |
| Diagnosis of UBPGL | Pre-surgical: a clear diagnosis of UBPGL before initial surgery; Post-surgical: a clear diagnosis of UBPGL after initial surgery | Pre-surgical/Post-surgical/not reported |
| Sweating | Excessive profuse sweating | Yes/no/not reported |
| Headache | Paroxysmal or sustained headache | Yes/no/not reported |
| Palpitations | Palpitations or reporting fast heartbeats | Yes/no/not reported |
| Hypertension | Known with history of hypertension or diagnosed hypertension before surgery defined as BP >140/90 or at antihypertension-medication | Yes/no/not reported |
| Nausea/vomiting | Nausea and/or vomiting | Yes/no/not reported |
| Hematuria | Hematuria (microscopic or macroscopic) | Yes/no/not reported |
| Other symptoms | Any other symptom believed to be related to the UBPGL | Free text |
| Catecholamine related Symptoms | Sweating, headache, palpitations or nausea/vomiting | Yes/no/not reported |
| Catecholamine related symptoms related to micturition | Catecholamine related symptoms elicited by micturition (during or after micturition) | Yes/no/not reported |
| Presurgical systolic BP | Highest systolic BP value before surgery (with or without details of measurement procedure) | mmHg |
| Presurgical diastolic BP | Highest diastolic BP value before surgery (with or without details of measurement procedure) | mmHg |
| Biopsy | Biopsy done before initial surgery. | Yes/no/not reported |
| Diagnosis of UBPGL by biopsy before surgery | Yes; UBPGL confirmed by pathology examination of biopsied tissue before surgery; no: if not a clear diagnosis of UBPGL according to the pathology of biopsy before surgery | Yes/no/not reported |
| Biochemical testing done before surgery | Any biochemical test (metanephrines, catecholamines and/or VMA of plasma and/or 24 hours urine) done before surgery | Yes/no/not reported |
| Result of biochemical testing | Yes: elevated result of any of biochemical tests; no: not elevated result of any of biochemical measurements | Yes/no |
| Alpha-adrenergic blockade before surgery | Yes; use of any alpha-adrenergic blocker as preparation for initial surgery; no: if not reported | Yes/no |
| Systolic BP during surgery | Maximal reported systolic BP during surgery | mmHg |
| Diastolic BP during surgery | Maximal reported diastolic BP during surgery | mmHg |
| Cardiovascular accident during surgery | Occurrence of any cardiovascular accident during surgery | Yes/no/not reported |
| Surgical procedure | Initial surgery approach, including patients in whom surgery had to be abandoned | TUR/Partial cystectomy/Radical cystectomy/not reported. |
| Discontinued surgery | Patients with surgery had to be interrupted and discontinued because of complications such as high blood pressure. | Yes/no |
| Residual tumor | Residual tumor after first surgery as verified by biochemical testing after surgery and/or by pathological evidence in case of additional surgery for the same tumor. | Yes/no/not reported |
| Additional surgery | Yes: if additional surgery was reported for patients with residual tumor or after discontinued surgery; no: if additional surgery was reported as not done for patients with residual tumor or discontinued surgery; or not reported | Yes/no/not reported |
| Surgical procedure of additional surgery | Additional surgery approach for patients with surgery discontinued or residual tumor | TUR/Partial cystectomy/Radical cystectomy/not reported. |
| Tumor size | Reported size according to imaging or pathology: if size was reported by less than three diameters, volume was calculated assuming the diameters were similar in all three directions. | cm^3^ |

M male, F female, UBPGL urinary bladder paraganglioma, BP blood pressure, TUR transurethral resection, VMA Vanillylmandelic acid.

**Table S2**. Papers and cases included.

| Paper No | Case No | Author | Journal | Year |
| --- | --- | --- | --- | --- |
| 1 | 1 | Jandou, I | Int J Surg Case Rep | 2020 |
| 2 | 2 | Teragaki, M | Intern Med | 2020 |
| 3 | 3 | Tan, J.L | ANZ J Surg | 2020 |
| 4 | 4 | Wang, S | Urol J | 2020 |
| 5 | 5 | Johnson, J.T | BMJ Case Rep | 2020 |
| 6 | 6 | Kazakova, V | BMJ Case Rep | 2020 |
| 7 | 7 | Rzepka, E | Endokrynol Pol | 2020 |
| 8 | 8 | Degrieck, B | J Belg Soc Radiol | 2020 |
| 9 | 9 | Choi, Y.H | Urology | 2020 |
| 10 | 10 | Sonmez, G | Urol Case Rep | 2020 |
| 11 | 11 | Yoo, K.H | Pathol Oncol Res | 2020 |
| 12 | 12 | Li, H | Mol Clin Oncol | 2020 |
|  | 13 | Li, H | Mol Clin Oncol | 2020 |
|  | 14 | Li, H | Mol Clin Oncol | 2020 |
|  | 15 | Li, H | Mol Clin Oncol | 2020 |
| 13 | 16 | Sunil, V.N | BMJ Case Rep | 2020 |
| 14 | 17 | Kurose, H | IJU Case Rep | 2020 |
| 15 | 18 | Alkhatatbeh, H | Urol Case Rep | 2020 |
| 16 | 19 | Cakici, M.C | J Coll Physicians Surg Pak | 2020 |
| 17 | 20 | Falcão, G | Pan Afr Med J | 2020 |
| 18 | 21 | Bosserman, A.J | Clin Nucl Med | 2019 |
| 19 | 22 | Chan, V | BMJ Case Rep | 2019 |
| 20 | 23 | Sugimura, R | IJU Case Rep | 2019 |
| 21 | 24 | Alanee, S | Urol Case Rep | 2019 |
| 22 | 25 | Roehmholdt, M | Curr Urol | 2019 |
| 23 | 26 | Białek, W | J Ultrason | 2019 |
| 24 | 27 | Hermi, A | Case Rep Urol | 2019 |
| 25 | 28 | Urabe, F | IJU Case Rep | 2019 |
| 26 | 29 | Malhotra, A.K | Can Urol Assoc J | 2018 |
| 27 | 30 | Chaaya, G | Am J Med Sci | 2018 |
| 28 | 31 | El-Tholoth, H.S | J Endourol Case Rep | 2018 |
| 29 | 32 | Kido, K | Transplant Proc | 2018 |
| 30 | 33 | Romano, I.J | J Am Soc Hypertens | 2018 |
| 31 | 34 | Kroiss, A.S | Rev Esp Med Nucl Imagen Mol | 2018 |
| 32 | 35 | Kaulanjan, K | J Endourol Case Rep | 2018 |
| 33 | 36 | Mahjoubi, Z | Urol Case Rep | 2018 |
| 34 | 37 | Kumar, A.V | Nephrology (Carlton) | 2017 |
| 35 | 38 | Lee, J | Diagn Cytopathol | 2017 |
| 36 | 39 | Youssef, A | Urol Case Rep | 2017 |
| 37 | 40 | Mithqal, A | Clin Nucl Med | 2017 |
| 38 | 41 | Lazareth, H | Mol Clin Oncol | 2017 |
| 39 | 42 | Fernandes, A.M | Radiol Bras | 2017 |
| 40 | 43 | El, A.A | BMJ Case Rep | 2017 |
| 41 | 44 | Wen, C.Y | Ci Ji Yi Xue Za Zhi | 2017 |
| 42 | 45 | Williams, P | JAAPA | 2017 |
| 43 | 46 | Hu, W | Int J Clin Exp Pathol | 2017 |
| 44 | 47 | Iwamoto, G | J Med Case Rep | 2017 |
| 45 | 48 | Ravichandran-Chandra, A | Cent European J Urol | 2017 |
| 46 | 49 | Yadav, S | BMJ Case Rep | 2016 |
| 47 | 50 | Spessoto, L.C | Urol Case Rep | 2016 |
| 48 | 51 | Gkikas, C | Urol Case Rep | 2016 |
| 49 | 52 | Yang, C | Oncol Lett | 2016 |
| 50 | 53 | Jain, T.K | World J Nucl Med | 2016 |
| 51 | 54 | Bishnoi, K | J Robot Surg | 2016 |
| 52 | 55 | Katiyar, R | J Clin Diagn Res | 2016 |
|  | 56 | Katiyar, R | J Clin Diagn Res | 2016 |
| 53 | 57 | Gupta, S | Endocr Pathol | 2016 |
| 54 | 58 | You, D | Mol Clin Oncol | 2016 |
| 55 | 59 | Marić, P | Vojnosanit Pregl | 2016 |
| 56 | 60 | Ching, D | Int J Surg Case Rep | 2016 |
| 57 | 61 | Salvatori, R | Endocrine | 2015 |
| 58 | 62 | Sangwatanaroj, S | Am J Med Sci | 2015 |
| 59 | 63 | Han, Y.J | Sao Paulo Med J | 2015 |
| 60 | 64 | Quist, E.E | Pathol Res Pract | 2015 |
| 61 | 65 | Verma, A | J Clin Diagn Res | 2015 |
| 62 | 66 | Shah, V.B | Indian J Pathol Microbiol | 2015 |
| 63 | 67 | Bagchi, A | Am J Case Rep | 2015 |
| 64 | 68 | Sherwani, P | Indian J Radiol Imaging | 2015 |
| 65 | 69 | Sajjan, R.S | Clin Nucl Med | 2015 |
| 66 | 70 | Peng, C | Oncol Lett | 2015 |
| 67 | 71 | Rayamajhi, S.J | Clin Nucl Med | 2015 |
| 68 | 72 | Priyadarshi, V | Urol Ann | 2015 |
| 69 | 73 | Valsangkar, R.S | Urol Ann | 2015 |
| 70 | 74 | Loveys, F.W | Radiographics | 2015 |
| 71 | 75 | Cai, Y | Medicine (Baltimore) | 2015 |
| 72 | 76 | Dattatrya, K.Y | J Clin Diagn Res | 2015 |
| 73 | 77 | Dragović, T | Vojnosanit Pregl | 2015 |
| 74 | 78 | Lacefield, E | Curr Urol | 2015 |
| 75 | 79 | Patnayak, R | J Cancer Res Ther | 2015 |
| 76 | 80 | Nerli, R.B | Indian J Surg Oncol | 2015 |
| 77 | 81 | Adraktas, D | Ultrasound Q | 2014 |
| 78 | 82 | Pichler, R | Urol Int | 2014 |
| 79 | 83 | Lai, Y | Oncol Lett | 2014 |
| 80 | 84 | Dhull, V.S | Clin Nucl Med | 2014 |
| 81 | 85 | Ranaweera, M | World J Clin Cases | 2014 |
|  | 86 | Ranaweera, M | World J Clin Cases | 2014 |
|  | 87 | Ranaweera, M | World J Clin Cases | 2014 |
| 82 | 88 | Neugarten, C.J | Urol Case Rep | 2014 |
| 83 | 89 | Calamaro, P | Anal Quant Cytopathol Histpathol | 2014 |
| 84 | 90 | Song, Y | Intern Med J | 2013 |
| 85 | 91 | Xiang, S.T | Chin Med J (Engl) | 2013 |
| 86 | 92 | Christodoulidou, M | BMJ Case Rep | 2013 |
| 87 | 93 | Beilan, J | BMC Res Notes | 2013 |
| 88 | 94 | Feng, N | Chin J Cancer | 2013 |
| 89 | 95 | Ahn, S.G | Can Urol Assoc J | 2013 |
| 90 | 96 | Maeda, M | Aktuelle Urol | 2013 |
| 91 | 97 | Kumar, U.M | J Clin Diagn Res | 2013 |
| 92 | 98 | Malik, A.A | BMJ Case Rep | 2013 |
| 93 | 99 | Li, S | Exp Ther Med | 2013 |
| 94 | 100 | Mallat, F | Case Rep Urol | 2013 |
| 95 | 101 | Li, W | Int J Clin Exp Med | 2013 |
| 96 | 102 | Khatavkar, S.S | J Anaesthesiol Clin Pharmacol | 2013 |
| 97 | 103 | Yang, Y | J Endourol | 2012 |
|  | 104 | Yang, Y | J Endourol | 2012 |
|  | 105 | Yang, Y | J Endourol | 2012 |
| 98 | 106 | Darlong, V | Singapore Med J | 2012 |
| 99 | 107 | Ghafoor, A.U | J Pak Med Assoc | 2012 |
| 100 | 108 | Manohar, K | Clin Nucl Med | 2012 |
| 101 | 109 | Hanji, A.M | Saudi J Kidney Dis Transpl | 2012 |
| 102 | 110 | Luchey, A | Can J Urol | 2012 |
| 103 | 111 | She, H.L | Ann Acad Med Singap | 2012 |
| 104 | 112 | El, K.F | J Med Liban | 2012 |
| 105 | 113 | Persec, Z | Coll Antropol | 2012 |
| 106 | 114 | Pahwa, H.S | BMJ Case Rep | 2012 |
| 107 | 115 | Wang, H | Eur J Radiol | 2011 |
|  | 116 | Wang, H | Eur J Radiol | 2011 |
|  | 117 | Wang, H | Eur J Radiol | 2011 |
|  | 118 | Wang, H | Eur J Radiol | 2011 |
| 108 | 119 | De Pasquale, V | Urol Int | 2011 |
| 109 | 120 | Bohn, O.L | Pediatr Dev Pathol | 2011 |
| 110 | 121 | Tsai, C.C | Kaohsiung J Med Sci | 2011 |
|  | 122 | Tsai, C.C | Kaohsiung J Med Sci | 2011 |
| 111 | 123 | Kang, S.G | Urol Int | 2011 |
| 112 | 124 | Bhalani, S.M | J Urol | 2011 |
| 113 | 125 | Zeitlin, I | Isr Med Assoc J | 2011 |
| 114 | 126 | Vyas, S | Indian J Nephrol | 2011 |
|  | 127 | Vyas, S | Indian J Nephrol | 2011 |
| 115 | 128 | Oderda, M | Scand J Urol Nephrol | 2010 |
| 116 | 129 | Pandey, R | Minerva Anestesiol | 2010 |
| 117 | 130 | Xu, D.F | J Med Case Rep | 2010 |
| 118 | 131 | Al-Zahrani, A.A | Adv Urol | 2010 |
| 119 | 132 | Nayyar, R | JSLS | 2010 |
| 120 | 133 | Schaefer, I.M | Cancer Genet Cytogenet | 2010 |
| 121 | 134 | Ghayee, H | Endocr Relat Cancer | 2009 |
| 122 | 135 | Cho, D.K | Eur Heart J | 2009 |
| 123 | 136 | Jayram, G | J Robot Surg | 2009 |
| 124 | 137 | Chen, Y.C | Kidney Int | 2009 |
| 125 | 138 | Huang, Y | Chin Med J (Engl) | 2009 |
| 126 | 139 | Mun, K.S | Malays J Pathol | 2009 |
| 127 | 140 | Yan, J.A | ANZ J Surg | 2009 |
| 128 | 141 | Chen, C.H | Can Urol Assoc J | 2009 |
| 129 | 142 | Tazi, M.F | Cases J | 2009 |
| 130 | 143 | Vesin, C | South Med J | 2009 |
| 131 | 144 | Mou, J.W | Pediatr Surg Int | 2008 |
| 132 | 145 | Kappers, M.H | Neth J Med | 2008 |
| 133 | 146 | Fournier, J.R | P R Health Sci J | 2008 |
| 134 | 147 | Heinrich, E | Urol J | 2008 |
| 135 | 148 | Im, S.H | Headache | 2008 |
| 136 | 149 | Dhawan, D.R | Urol J | 2008 |
| 137 | 150 | Sharma, P.K | Indian J Surg | 2008 |
| 138 | 151 | Havekes, B | J Clin Endocrinol Metab | 2007 |
| 139 | 152 | Madani, R | World J Surg | 2007 |
|  | 153 | Madani, R | World J Surg | 2007 |
| 140 | 154 | Huang, K.H | Int J Urol | 2007 |
| 141 | 155 | Kairi-Vassilatou, E | Eur J Gynaecol Oncol | 2007 |
| 142 | 156 | Sharma, S | Indian J Surg | 2007 |
| 143 | 157 | Thambugala, G.M | Australas Radiol | 2007 |
| 144 | 158 | Athyal, R.P | Australas Radiol | 2007 |
| 145 | 159 | Safwat, A.S | Can J Urol | 2007 |
|  | 160 | Safwat, A.S | Can J Urol | 2007 |
|  | 161 | Safwat, A.S | Can J Urol | 2007 |
| 146 | 162 | Dilbaz, B | Surg Laparosc Endosc Percutan Tech | 2006 |
| 147 | 163 | Bozbora, A | JSLS | 2006 |
| 148 | 164 | Tsutsui, A | Hinyokika Kiyo | 2006 |
| 149 | 165 | Segawa, N | Hinyokika Kiyo | 2005 |
| 150 | 166 | Purandare, N.C | J Ultrasound Med | 2005 |
| 151 | 167 | Naqiyah, I | Singapore Med J | 2005 |
| 152 | 168 | Usuda, H | Pathol Int | 2005 |
| 153 | 169 | Kovacs, K | Endocr Pathol | 2005 |
| 154 | 170 | Yoshida, S | Int J Urol | 2004 |
| 155 | 171 | Pastor-Guzmán, J.M | Urol Int | 2004 |
|  | 172 | Pastor-Guzmán, J.M | Urol Int | 2004 |
| 156 | 173 | Minagawa, T | Hinyokika Kiyo | 2004 |
| 157 | 174 | Lawrence, J.K | Hormones (Athens) | 2004 |
| 158 | 175 | Hwang, J.J | J Urol | 2003 |
| 159 | 176 | Kang, W.Y | Kaohsiung J Med Sci | 2003 |
| 160 | 177 | Onishi, T | Int J Urol | 2003 |
| 161 | 178 | Dundr, P | Pathol Res Pract | 2003 |
| 162 | 179 | Naguib, M | Am J Clin Oncol | 2002 |
| 163 | 180 | Peczkowska, M | Blood Press | 2002 |
| 164 | 181 | Nakatani, T | Oncol Rep | 2002 |
| 165 | 182 | Lamarre-Cliche, M | Circulation | 2002 |
| 166 | 183 | Tan, S.M | Anaesth Intensive Care | 2002 |
| 167 | 184 | Doran, F | APMIS | 2002 |
| 168 | 185 | Gaur, D.D | Minim Invasive Ther Allied Technol | 2002 |
| 169 | 186 | Seki N | Urol Int | 2001 |
| 170 | 187 | Kozlowski, P.M | Urology | 2001 |
| 171 | 188 | Takezawa, Y | Hinyokika Kiyo | 2001 |
| 172 | 189 | Salanitri, J | Australas Radiol | 2001 |
| 173 | 190 | Moritani, H | Intern Med | 2001 |
| 174 | 191 | Dewan, M | J R Soc Promot Health | 2001 |
| 175 | 192 | Taue, R | Int J Urol | 2001 |
| 176 | 193 | Bonacruz, K.G | J Paediatr Child Health | 2001 |
| 177 | 194 | Ansari, M.S | Int Urol Nephrol | 2001 |

**Table S3.** Clinical features of patients with pre- or postsurgical diagnosis of urinary bladder paraganglioma.

|  | Total | Presurgical diagnosis | | Postsurgical diagnosis | | P value | |
| --- | --- | --- | --- | --- | --- | --- | --- |
| Patients, n | 194 | 90 | 104 | |  | |  |
| Females, n (%)^*^ | 98 (50.5) | 44 (48.9) | 54 (51.9) | | 0.396 | |  |
| Age, mean± SD (years) | 43.6± 18.6 | 39.7± 18.7 | 47.1± 17.9 | | 0.005 | |  |
| Presurgical SBP, mean± SD (mmHg)^$^ | 169.2± 47.9 | 184.7± 45.8 | 138± 31 | | <0.001 | |  |
| Presurgical DBP, mean± SD (mmHg)^$$^ | 100.9± 25.2 | 108.9± 23.5 | 85.8± 18.9 | | <0.001 | |  |
| Primary tumor, n (%) | 183 (94.3) | 82 (91.1) | 101 (97.1) | | 0.117 | |  |
| Signs and symptoms, n (%) |  |  |  | |  | |  |
| - Hypertension | 95 (49.0) | 68 (75.6) | 27 (26.0) | | <0.001 | |  |
| - Catecholamine-associated symptoms^#^ | 87 (44.8) | 61 (67.8) | 26 (25.0) | | <0.001 | |  |
| - Hematuria | 67 (34.5) | 21 (23.3) | 46 (44.2) | | 0.003 | |  |
| - Other | 22 (11.3) | 5 (5.6) | 17 (16.3) | | 0.022 | |  |
| - None | 20 (10.3) | 3 (3.3) | 17 (16.3) | | 0.004 | |  |
| Catecholamine-associated symptoms, n (%) |  |  |  | |  | |  |
| - Sweating | 25 (12.9) | 20 (22.2) | 5 (4.8) | | <0.001 | |  |
| - Headache | 63 (32.5) | 49 (54.4) | 14 (13.5) | | <0.001 | |  |
| - Palpitations | 47 (24.2) | 35 (38.9) | 12 (11.5) | | <0.001 | |  |
| - Nausea/vomiting | 8 (4.1) | 6 (6.7) | 2 (1.9) | | 0.148 | |  |
| Catecholamine-associated symptoms triggered by  micturition, n (%) | 59 (30.4) | 46 (51.1) | 13 (12.5) | | <0.001 | |  |
| Alpha-adrenergic blockade before initial surgery, n (%) | 53 (27.3) | 48 (53.3) | 5 (4.8) | | <0.001 | |  |
| Initial surgical approach, n (%) |  |  |  | | <0.001 | |  |
| - TUR | 78 (40.2) | 5 (5.6) | 73 (70.2) | |  | |  |
| - Partial cystectomy | 87 (44.8) | 65 (72.2) | 22 (21.2) | |  | |  |
| - Radical cystectomy | 8 (4.1) | 6 (6.7) | 2 (1.9) | |  | |  |
| - Not reported | 21 (10.8) | 14 (15.6) | 7 (6.7) | |  | |  |
| Patients with SBP >180 mmHg during surgery, n (%) | 28 (14.4) | 3 (3.3) | 25 (24) | | <0.001 | |  |
| - without reported presurgical hypertension | 20 (71.4) | 1 (33.3) | 19 (76) | |  | |  |
| - with reported presurgical hypertension | 8 (28.6) | 2 (66.7) | 6 (24) | |  | |  |
| Size (cm), median (IQR)^&^  Cardiovascular incidents during surgery, n (%) | 3 (2.3-4.3)  10 (5.2) | 3.7 (2.6-5.0)  0 | 2.7 (2.0-3.7)  10 (9.6) | | <0.001  0.002 | |  |
| Discontinued surgery, n (%) | 14 (7.2) | 1 (1.1) | 13 (12.5) | | 0.004 | |  |
| Residual tumor, n (%) | 33 (17.0) | 5 (5.6) | 28 (26.9) | | <0.001 | |  |

UBPGL urinary bladder paraganglioma, SBP systolic blood pressure, DBP diastolic blood pressure, TUR transurethral resection,

IQR interquartile ranges.

^*^ In two patients gender was not reported.

^$^ In 43 presurgical diagnosis and 80 postsurgical diagnosis patients SBP was not reported.

^$$^ In 48 presurgical diagnosis and 80 postsurgical diagnosis patients DBP was not reported.

^#^ Patients presented with symptoms (single or multiple) in sweating, headache, palpitation or nausea/vomiting.

^&^ Tumor size of 23 patients with presurgical diagnosis and 24 patients with postsurgical diagnosis of UBPGL were not available.

**Table S4.** Characteristics of patients with positive or negative biochemical testing results versus without biochemical testing.

|  | Without  biochemical test | With  biochemical test | |
| --- | --- | --- | --- |
|  |  | Positive  results | Negative  results |
| Patients, n | 90 | 82 | 22 |
| Alpha-adrenergic blockade before surgery, n (%) | 3 (3.3) | 45 (54.9)^**^ | 5 (22.7)^$^ |
| Initial surgical approach, n (%) |  |  |  |
| -       TUR | 65 (72.2) | 5 (6.1)^**^ | 8 (36.4)^$^ |
| -       Cystectomy | 21 (23.3) | 65 (79.3)^**^ | 9 (40.9) |
| -       Not clear | 4 (4.4) | 12 (14.6)^*^ | 5 (22.7)^$^ |
| Patients with SBP>180 mmHg during surgery, n (%) | 20 (22.2) | 3 (3.7)^**^ | 5 (22.7) |
| Cardiovascular incidents during surgery, n (%) | 10 (11.1) | 0^*^ | 0 |
| Discontinued surgery, n (%) | 12 (13.3) | 1 (1.2)^*^ | 1 (4.5) |
| Residual tumor, n (%) | 25 (27.8) | 5 (6.1)^**^ | 3 (13.6) |

TUR transurethral resection, SBP systolic blood pressure

^*^ P<0.05, ^**^ P<0.001, indicating the differences between patients with positive biochemical testing results versus without biochemical testing.

^$^ P<0.05, indicating the differences between patients with negative biochemical testing results versus without biochemical testing.

**Table S5.** Univariable and multivariable binary logistic regression analysis of factors associated with a probability of presurgical biochemical testing.

|  | Univariable logistic regression | Multivariable logistic regression |
| --- | --- | --- |
| Age (year) | 0.98 (0.96-1.00)^*^ | 0.97 (0.94-0.99)^*^ |
| Size (cm)^#^ | 1.35 (1.09-1.68)^*^ | 1.13 (0.85-1.49) |
| Signs and symptoms |  |  |
| - Hypertension | 5.92 (3.17-11.04)^**^ | 4.45 (1.66-11.94)^*^ |
| - Catecholamine-associated symptoms | 7.06 (3.69-13.49)^**^ | 2.33 (0.66-8.18) |
| - Hematuria | 0.27 (0.14-0.50)^**^ | 0.23 (0.09-0.60)^*^ |
| - Other | 0.28 (0.11-0.76)^*^ | 0.45 (0.09-2.11) |
| - None | 0.68 (0.27-1.73) |  |
| Catecholamine-associated symptoms triggered by micturition | 8.33 (3.79-18.34)^**^ | 2.14 (0.53-8.65) |

Results were presented as OR (95% CI).

^#^ There were 47 missing values.

^*^ P<0.05; ^**^ P<0.001

**Figure S1.**


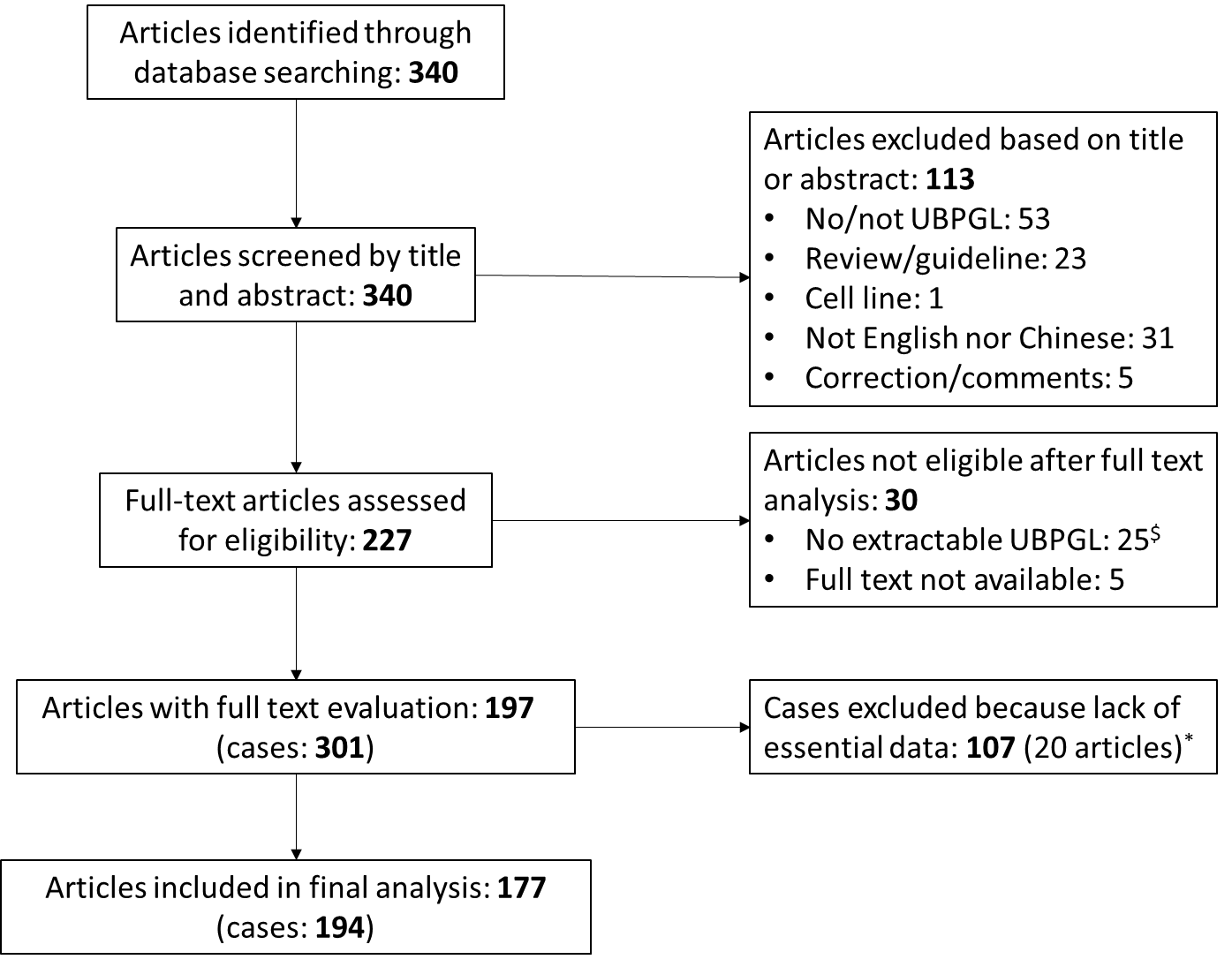


**Figure S2.**


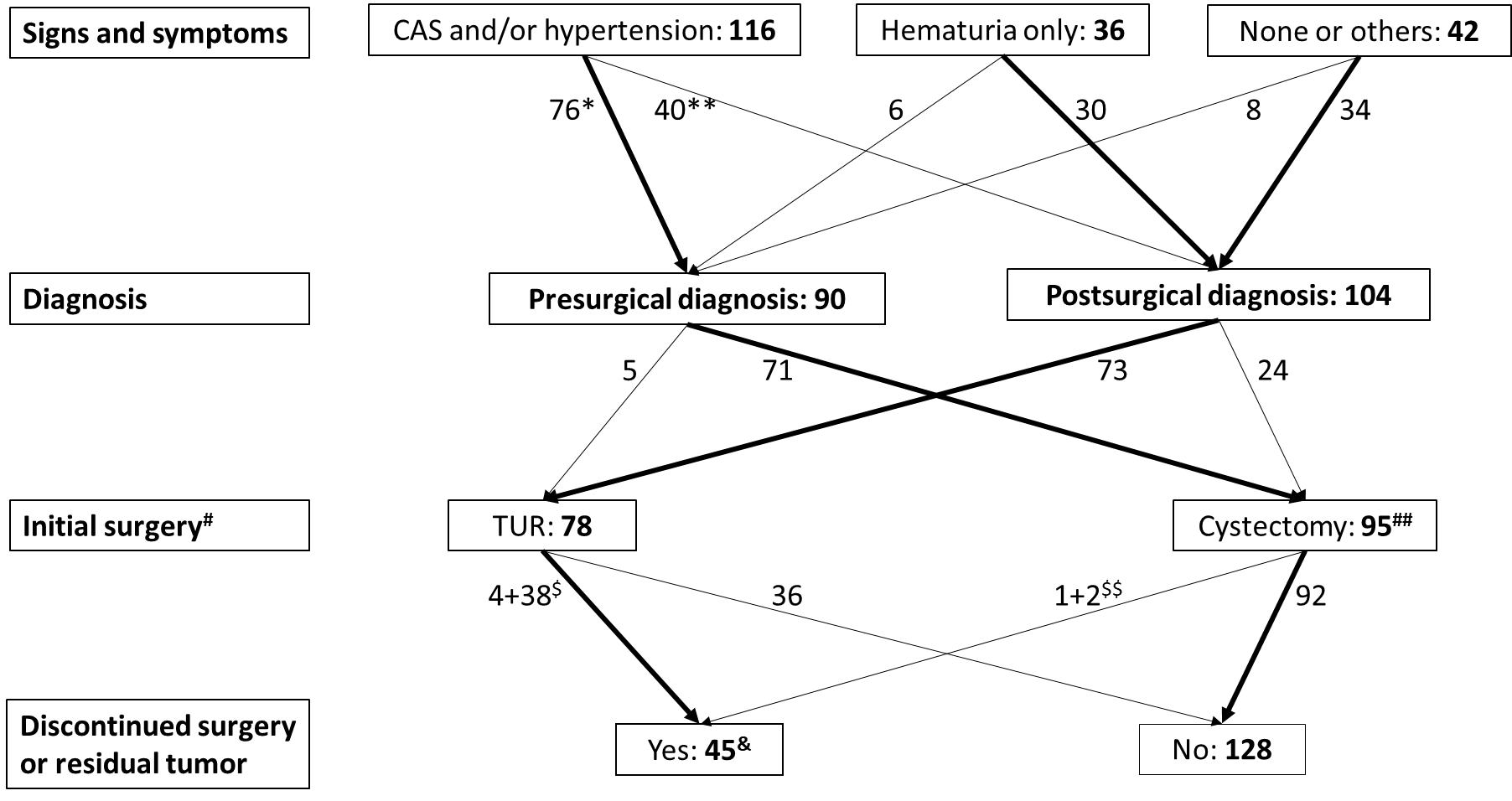


**Figure S3.**


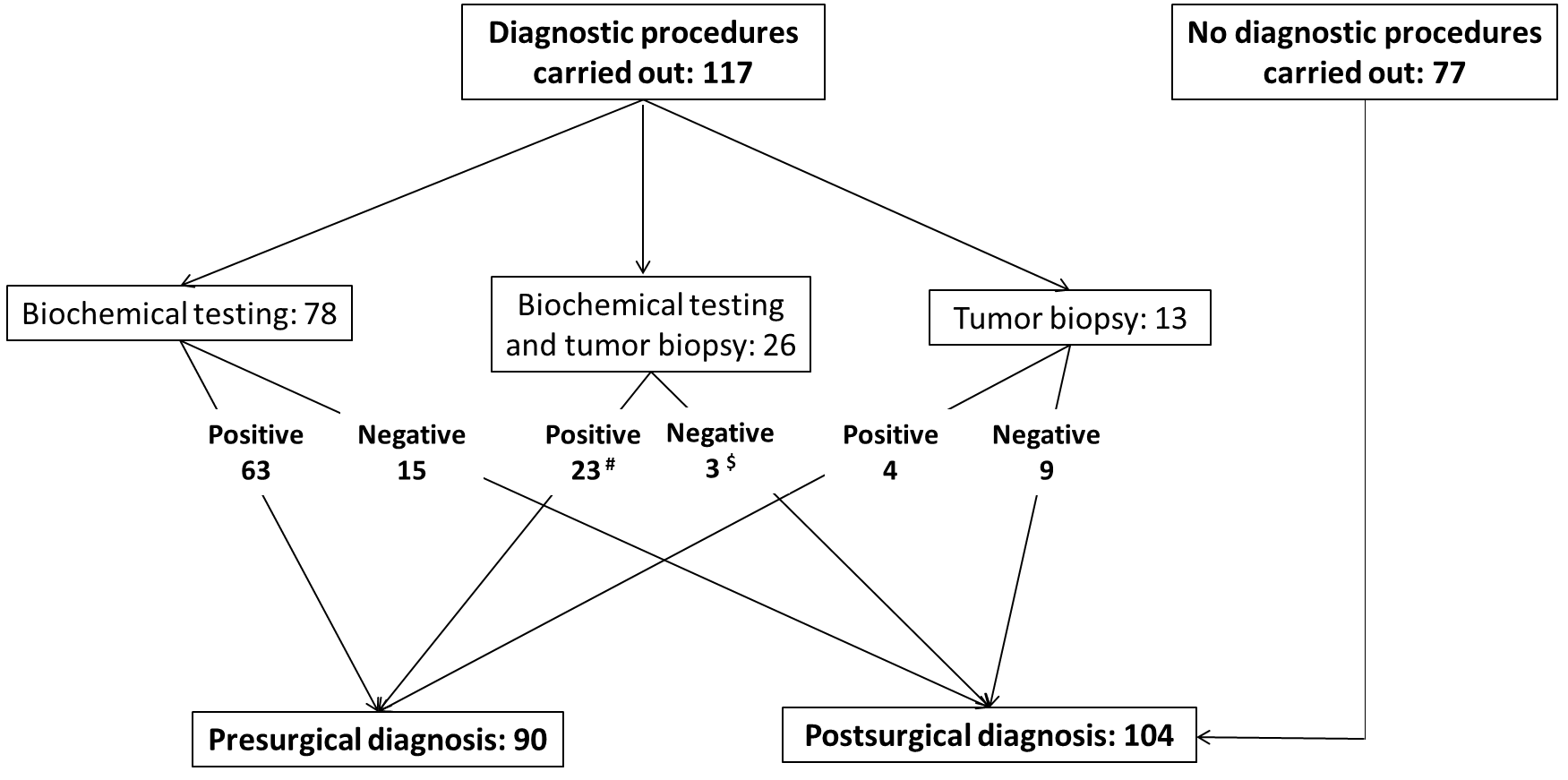


**Figure legends**

**Figure S1:** **Flow chart of studies considered for analysis.**

Some articles contained more than one case.

^$^ Twenty-five articles were excluded from further analysis because they provided no detailed description of individual clinical data in single patients.

^*^ Twenty articles were excluded from the analysis because the times of diagnosis (before or after surgery) of the urinary bladder paraganglioma (UBPGL) was not reported.

**Figure S2: Flow diagram of the number of patients in different phases of clinical management.**

CAS, catecholamine-associated symptoms.

^*^ Including 15 patients with hematuria; ^**^ Including 16 patients with hematuria.

^#^ Initial surgical approach was not available in 21 patients; ^##^ Including laparoscopic or open partial cystectomy for 87 patients and radical cystectomy for eight patients.

^$^ Four patients were presurgically diagnosed and 38 patients were postsurgically diagnosed; ^$$^ One patient was presurgically diagnosed and two patients were postsurgicallly diagnosed.

^&^ Patients with discontinued surgery (n=13) or with residual tumor (n=32) after surgery.

**Figure S3: Flow diagram of diagnostic procedures related to the time of diagnosis.**

# Patients who had positive results of biochemical testing and/or tumor biopsy.

$ Patients who had negative results of biochemical testing and tumor biopsy.
